# Cardiac Per2 Functions as Novel Link between Fatty Acid Metabolism and Myocardial Inflammation during Ischemia and Reperfusion Injury of the Heart

**DOI:** 10.1371/journal.pone.0071493

**Published:** 2013-08-20

**Authors:** Stephanie Bonney, Doug Kominsky, Kelley Brodsky, Holger Eltzschig, Lori Walker, Tobias Eckle

**Affiliations:** 1 Department of Anesthesiology and Mucosal Inflammation Program, University of Colorado Denver, Aurora, Colorado, United States of America; 2 Division of Cardiology, Department of Medicine, University of Colorado Denver, Aurora, United States of America; University of Tübingen, Germany

## Abstract

Disruption of peripheral circadian rhyme pathways dominantly leads to metabolic disorders. Studies on circadian rhythm proteins in the heart indicated a role for Clock or Per2 in cardiac metabolism. In contrast to *Clock^−/−^*, *Per2^−/−^* mice have larger infarct sizes with deficient lactate production during myocardial ischemia. To test the hypothesis that cardiac Per2 represents an important regulator of cardiac metabolism during myocardial ischemia, we measured lactate during reperfusion in *Per1^−/−^, Per2^−/−^* or wildtype mice. As lactate measurements in whole blood indicated an exclusive role of Per2 in controlling lactate production during myocardial ischemia, we next performed gene array studies using various ischemia-reperfusion protocols comparing wildtype and *Per2^−/−^* mice. Surprisingly, high-throughput gene array analysis revealed dominantly lipid metabolism as the differentially regulated pathway in wildtype mice when compared to *Per2^−/−^*. In all ischemia-reperfusion protocols used, the enzyme enoyl-CoA hydratase, which is essential in fatty acid beta-oxidation, was regulated in wildtype animals only. Studies using nuclear magnet resonance imaging (NMRI) confirmed altered fatty acid populations with higher mono-unsaturated fatty acid levels in hearts from *Per2^−/−^* mice. Unexpectedly, studies on gene regulation during reperfusion revealed solely pro inflammatory genes as differentially regulated ‘Per2-genes’. Subsequent studies on inflammatory markers showed increasing IL-6 or TNFα levels during reperfusion in *Per2^−/−^* mice. In summary, these studies reveal an important role of cardiac Per2 for fatty acid metabolism and inflammation during myocardial ischemia and reperfusion, respectively.

## Introduction

Epidemiological studies have shown that shift or nighttime workers have a higher incidence of cardiovascular disease [1]–[7]. Because blood pressure [8], heart rate [9], endothelial function [10], and the onset of myocardial infarction and stroke [10], [11] have distinct circadian patterns, it has been suggested that disruption of circadian rhythms may contribute to cardiovascular disease [12]. The primary control of the circadian clock is located in the hypothalamic suprachiasmatic nuclei (SCN), which are used to synchronize peripheral clocks in organs and tissues [13], [14]. This synchronization is accomplished through a series of tightly regulated circadian genes such as Clock, Brain and muscle Arnt-like protein-1 (Bmal1)[15], Neuronal PAS domain protein 2 (Npas2), Cryptochrome (Cry1/2), and Period (Per1/2). Mutations in or knockout of these circadian genes lead to diverse pathophysiological disorders, including metabolic syndrome, obesity [16], premature aging [17], and abnormal sleep cycle [18]. Interestingly, the circadian clock in metabolism is one of the most studied areas in the field, outside of the central clock [19]. For example, *Bmal1^−/−^* and *Clock^−/−^* mice are diabetic [20]. *Clock^−/−^* mice display a metabolic syndrome [16] and *Cry1^−/−^* mice develop hypertension [21]. Cardiomyocyte-specific circadian Clock mutant (CCM) mice reveal Clock as the direct regulator of triglyceride metabolism in the heart [22] and an adipocyte-specific deletion of Arntl in mice results in obesity [23], which is associated with a reduced number of polyunsaturated fatty acids in adipocyte triglycerides. Recent studies implicated Per2 in the regulation of fatty acid metabolism with increased oxygen consumption in *Per2^−/−^* mice [24]. Our group found impaired glycolysis during myocardial ischemia and severe depletion of glycogen storages leading to dramatically increased infarct sizes in *Per2^−/−^* mice [25]. Based on these findings we hypothesized an important role of Per2 in regulating cardiac metabolism. To gain insight into innate cardiac Per2 mediated adaptive mechanisms during myocardial ischemia we performed a detailed microarray analysis using different ischemia and reperfusion protocols. Here, we found a novel role of Per2 in controlling fatty acid metabolism and inflammation during ischemia and reperfusion, respectively.

## Materials and Methods

### Mouse Experiments

Experimental protocols were approved by the Institutional Review Board (IRB) at the University of Colorado Denver, USA. They were in accordance with the NIH guidelines for use of live animals. Before experiments, mice were housed for at least 4 weeks in a 14/10-h light-dark cycle to synchronize (entrain) the circadian clock of WT mice to the ambient light-dark cycle. We conducted all mouse experiments at same time points (ZT 0). To eliminate gender or age-related variations, we used 12- to 16-week-old male mice.

### *Per2^−/−^* mice

*Per2^−/−^* mice were obtained from the Jackson Laboratories [26]. Characterization and validation was performed as described previously. Dr. Cheng-Chi Lee kindly provided the *Per1^−/−^* mice [27]. Homozygous mutant mice are morphologically indistinguishable from their wild-type littermates and both males and females are fertile.

### Murine Model for cardiac ischemia

Anesthesia was induced (70 mg/kg body weight i.p.) and maintained (10 mg/kg/h) with sodium pentobarbital. Mice were placed on a temperature-controlled heated table (RT, Effenberg, Munich, Germany) with a rectal thermometer probe attached to a thermal feedback controller to maintain body temperature at 37°C. The tracheal tube was connected to a mechanical ventilator (Servo 900C, Siemens, Germany) with pediatric tubing and the animals were ventilated with a pressure controlled ventilation mode (peak inspiratory pressure of 10 mbar, frequency 110 breaths/min, positive end-expiratory pressure of 3 mbar, FiO_2_  = 0.3). Blood gas analysis revealed normal paO_2_ (115±15 mmHg) and paCO_2_ (38±6 mmHg) levels with our ventilator regime. After induction of anesthesia, animals were monitored with a surface electrocardiogram (ECG, Hewlett Packard, Böblingen, Germany). Fluid replacement was performed with normal saline, 0.2 ml/h i.v. The carotid artery was catheterized for continuous recording of blood pressure with a statham element (WK 280, WKK, Kaltbrunn, Switzerland). Operations were performed under an upright dissecting microscope (Olympus SZX12). Following left anterior thoracotomy, exposure of the heart and dissection of the pericardium, the left coronary artery (LCA) was visually identified and an 8.0 nylon suture (Prolene, Ethicon, Norderstedt, Germany) was placed around the vessel. Atraumatic LCA occlusion for ischemia or IP studies was performed using a hanging weight system [28]–[32]. Successful LCA occlusion was confirmed by an immediate color change of the vessel from light red to dark violet, and of the myocardium supplied by the vessel from bright red to white, as well as the immediate occurrence of ST-elevations in the ECG. During reperfusion, the changes of color immediately disappeared when the hanging weights were lifted and the LCA was perfused again [33]–[35].

### Lactate measurements and blood gas analysis

To determine lactate from whole blood samples, arterial blood was obtained via cardiac puncture and samples were analyzed immediately after collection with the I-STAT Analyzer (Abbott) [36].

### Microarray analysis

Ischemia (30 minutes, I30), ischemic preconditioning (IP0, 4 cycles of 5 min ischemia and 5 min reperfusion) and ischemia with reperfusion (30 min ischemia and 60 minutes reperfusion, I30R60) was performed in C57BL6 (The Jackson Laboratory) and *Per2^−/−^* mice. Heart tissue was snap-frozen with clamps pre-cooled to the temperature of liquid nitrogen. Total RNA was isolated from preconditioned heart tissue with the RNeasy micro kit (Qiagen, Valencia, CA) using Qiagen on-column DNase treatment to remove any contaminating genomic DNA. The integrity of RNA was assessed using an Agilent 2100 Bioanalyzer (Agilent Technologies) and RNA concentration was determined using a NanoDrop ND-1000 spectrophotometer (NanoDrop, Rockland, DE). Biotinylated cRNA were prepared according to the standard Affymetrix protocol from 150ng total RNA (Expression Analysis Technical Manual, 2001, Affymetrix). Following fragmentation, 10 µg of cRNA were hybridized for 16 h at 45 C on GeneChip Mouse Gene 1.1 ST Array Plate. GeneChips were washed and stained in a GeneTitan® Multi-Channel (MC) Instrument. Each array was subjected to visual inspection for gross abnormalities. Several other QC metrics were used to monitor hybridization efficiency and RNA integrity over the entire processing procedure. Raw image files were processed using Affymetrix GCOS 1.3 software to calculate individual probe cell intensity data and generate CEL data files. Using GCOS and the MAS 5.0 algorithm, intensity data was normalized per chip to a target intensity TGT value of 500 and expression data and present/absent calls for individual probe sets calculated. Quality control was performed by examining raw DAT image files for anomalies, confirming each thtat GeneChip array had a background value less than 100, monitoring that the percentage present calls was appropriate for the cell type, and inspecting the poly (A) spike in controls, housekeeping genes, and hybridization controls to confirm labeling and hybridization consistency. For each array, CEL files were imported into *Partek Genomic Suite software 6.6* (Partek), and data were normalized using the RMA (Robust Multichip Averaging) algorithm. According to our experimental setup the arrays were normalized, grouped and analyzed for differentially expressed transcripts based on different statistical tests. Using the “*Ingenuity Pathway Analysis*” we were able to identify biological mechanisms, pathways and functions most relevant to our experimental dataset. The array data has been deposited at Gene Expression Omnibus (GEO) repository at the National Center for Biotechnology Information (NCBI) GEO, accession number GSE45818, and is accessible through the following online link: http://www.ncbi.nlm.nih.gov/geo/query/acc.cgi?token=vdmvhysewqueudg&acc=GSE45818.

### Metabolic NMR

In order to determine fatty acid populations, *Per2^−/−^* mice or littermate controls matched in age, weight and gender were exposed to 30 min of in situ myocardial ischemia. With a loop suture in place, whole hearts were snap-frozen with clamps pre-cooled to the temperature of liquid nitrogen. *Extraction Protocols for Metabolic NMR.* Collected frozen heart specimens were homogenated in ice-cold 8% perchloric acid (PCA) as described previously. Briefly, after centrifugation, the supernatants (containing hydrophilic metabolites) were collected and pH was adjusted to pH = 7 using KOH. The potassium perchlorate was removed by centrifugation, and the hydrophilic fraction was lyophilized overnight. The tissue pellets (after the first centrifugation), which contained the lipophilic metabolites, were re-dissolved in water and pH was adjusted (7.0). The lipophilic fraction was lyophilized overnight. The dried hydrophilic tissue extracts were re-dissolved in 0.5 mL of deuterium oxide (D2O), transferred into 5-mm NMR tubes and used for 1H- and 31P-NMR analysis. The tissue lipid extracts were re-dissolved in 1.2 mL of deuterated chloroform/deuterated methanol mixture (2:1 vol/vol) [37]. *NMR Analysis on Tissue Extracts*. All 1H-NMR spectra were obtained at the Bruker 500 MHz DRX NMR spectrometer using an inverse Bruker 5-mm TXI probe. All spectra were Fourier transformed and lactate (Lac3, CH3) was used as an internal chemical reference (1.32 ppm). For metabolite quantification, one dimensional 1H-NMR spectra were obtained from each sample, with a standard water pre-saturation pulse program “zgpr”. A thin sealed glass capillary, containing TSP, was placed in each 5-m m tube prior to 1H-NMR experiments. The total number of acquisitions varied from 40 to 128. Conventional 1H acquisition parameters were: power level pl1 = 20dB; power angle p1 = 6.3 sec (90 degree pulse); power level for water pre-saturation pl9 = 77 dB; water suppression at O1P = 4.76 ppm; spectral width SW = 5000 MHz; and the pulse delay of 12.75 s (calculated as 5*T1) was applied between acquisitions for fully relaxed 1H-NMR spectra. The TSP from reference capillary served as a chemical shift (0 ppm) and proton metabolite concentration reference.

Before 31P-NMR analysis, 100 mmol/L EDTA was added to the tissue extracts to complex divalent cations. All 31P-NMR spectra (with proton decoupling) on cell extracts were obtained at the Bruker 300 MHz Avance NMR spectrometer using a Bruker QNP probe. The total number of scans was 12,000 per extract. A thin capillary glass containing 2.3 mmol/L methyl-diphosphoric acid (MDPA) was placed in each 5-mm NMR tube and serve as a chemical shift (18.6 ppm) and phosphor metabolite concentration reference. To calculate an absolute monounsaturated fatty acid (MUFA) concentration, the concentration of polyunsaturated fatty acids, triacylglycerides and glycerides are subtracted from the total for this peak.

### ELISA (IL-6, TNF-α) from heart tissue

The snap-frozen hearts were thawed, weighed, transferred to different tubes on ice containing 1 ml of Tissue Protein Extraction Reagent (T-PER; Pierce Biotechnology). Tissues were homogenized at 4°C. Homogenates were centrifuged at 9,000 g for 10 min at 4°C. Supernatants were transferred to clean microcentrifuge tubes, frozen on dry ice, and thawed on ice. Total protein concentrations in the tissue homogenates were determined using a bicinchoninic acid kit (Pierce Biotechnology). IL-6 (R&D Systems) or, TNF-α (R&D Systems) tissue concentrations were evaluated using a mouse ELISA kit according to the user's manual.

### Data analysis

Data were compared by two-factor ANOVA with Bonferroni's post-hoc test, or by Student's t-test where appropriate. Values are expressed as mean (SD) from 3 animals per condition. The chosen numbers of animals per group was based on findings in previous studies and a subsequent samples size analysis. The studies are designed to be able to reject the null hypothesis that the population means of the experimental and control groups are equal with probability (power) 0.8. The Type I error probability associated with this test of this null hypothesis is 0.05. *P*<0.05 was considered statistically significant. For all statistical analysis, GraphPad Prism 5.0 software for Windows was used. The authors had full access to and take full responsibility for the integrity of the data. All authors have read and agree to the manuscript as written.

## Results

### Per2 but not Per1 dependent lactate production during myocardial ischemia

**Figure 1:**
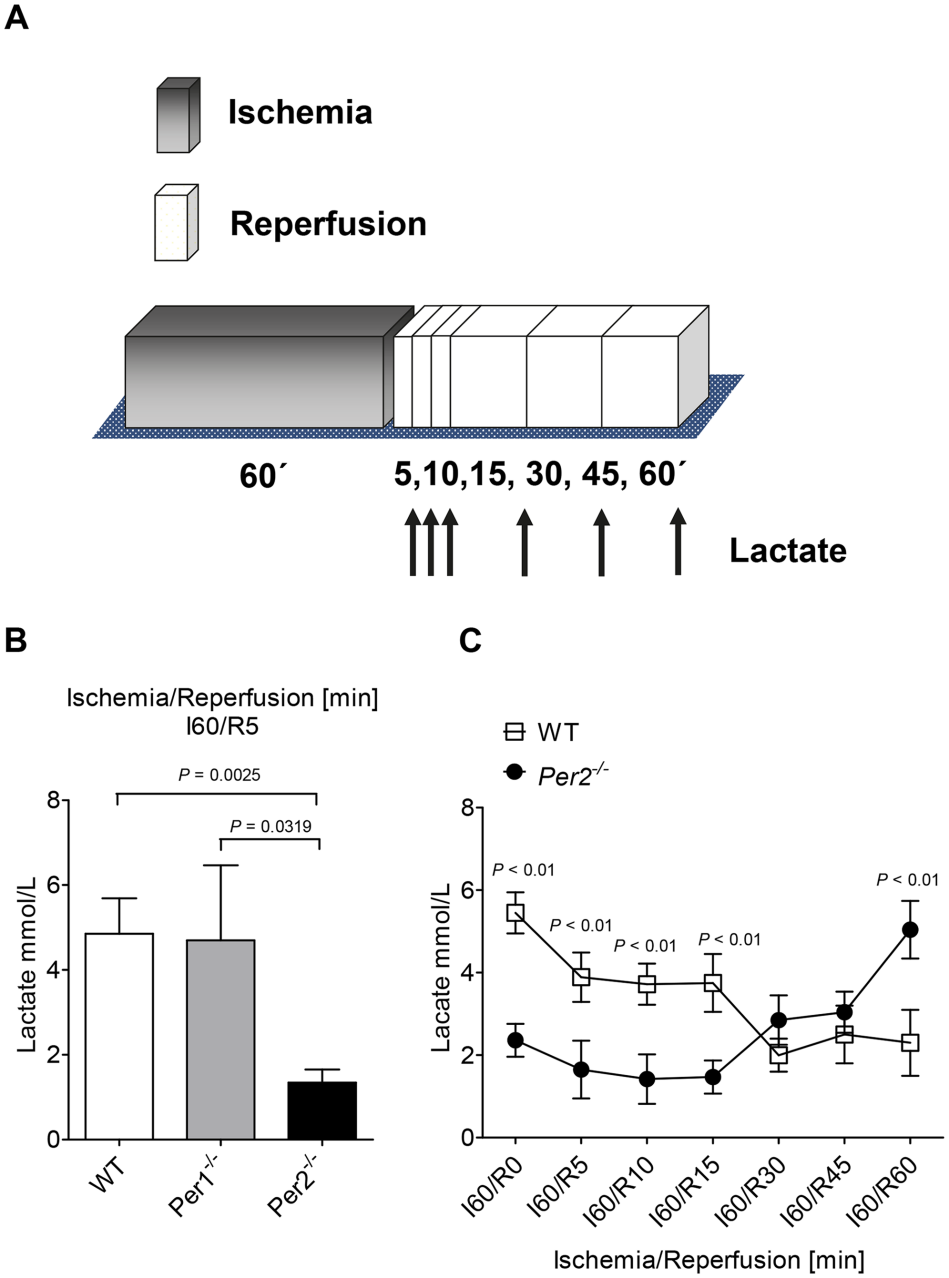
Lactate measurements from whole blood in wildtype, *Per1* *^−^* ^*/−*^ and *Per2* *^−^* ^*/−*^ mice. (**A**) Murine model of in situ myocardial ischemia and reperfusion. After 60 minutes of ischemia and indicated time points of reperfusion whole blood samples were obtained by left ventricular puncture. (**B**) Lactate measurements in wildtype (WT), Period 1 deficient (*Per1^−/−^*) and Period 2 deficient (*Per2^−/−^*) mice after 60 minutes of ischemia and 5 minutes of reperfusion. (C) Time course of lactate levels in whole blood after 60 minutes of ischemia and indicated time points of reperfusion (0, 5, 10, 15, 30, 45 and 60 minutes) in wildtype and *Per2^−/−^* mice; n = 3 mice in all groups.

Recent studies found a lack of lactate production in *Per2^−/−^* mice during myocardial ischemia, which was associated with larger infarct sizes after 60 minutes of ischemia. In these studies, lactate levels were determined after the infusion of labeled glucose using mass spectrometry in tandem with high-performance liquid chromatography [25]. Here we thought to confirm these findings using a clinically relevant and more convenient device. As lactate levels rise in the circulation immediately after the onset of reperfusion, we determined lactate levels in whole blood samples using the I-STAT [36]. After 60 minutes of ischemia and indicated time-periods of reperfusion (**Figure** **1A** **)**, we collected whole blood samples by left ventricular puncture. As shown in **Figure** **1B**, wildtype animals reached lactate levels of 4.85 mmol/L (SD0.8) after 5 minutes of reperfusion. Although recent studies analyzed infarct sizes *in Per1^−/−^* mice [25], indicating that the observed cardiac phenotype in *Per2^−/−^* was indeed Per2 specific, no lactate levels were determined in *Per1^−/−^*. Therefore, we next determined lactate levels in *Per1^−/−^* mice to evaluate a potential role of Per1 for cardiac carbohydrate metabolism. This showed similar lactate levels in *Per1^−/−^* as in wildtype mice (4.7 mmol/L (SD2.7)). However, following studies in *Per2^−/−^*, there was no increase in lactate levels when compared to wildtype or *Per1^−/−^* mice (**Figure** **1B**, 1.34 mmol/L (SD0.4), *P* = 0.0025 over wildtype, n = 3). Interestingly, while wildtype animals metabolized lactate to baseline levels within 30 minutes of reperfusion, *Per2^−/−^* started lactate production, which led to significantly higher levels after 60 minutes of reperfusion (**Figure** **1C** **,** wildtype: 2.3 mmol/L (SD0.8) vs. *Per2^−/−^*:5.04 mmol/L (SD0.7), * *P*<0.001, n = 3). Taken together, these data confirm a dominant role of Per2 for lactate production during myocardial ischemia and suppose different roles for Per2 during ischemia or reperfusion.

### High throughput gene array analysis from *Per2^−/−^* and wildtype hearts

**Figure 2:**
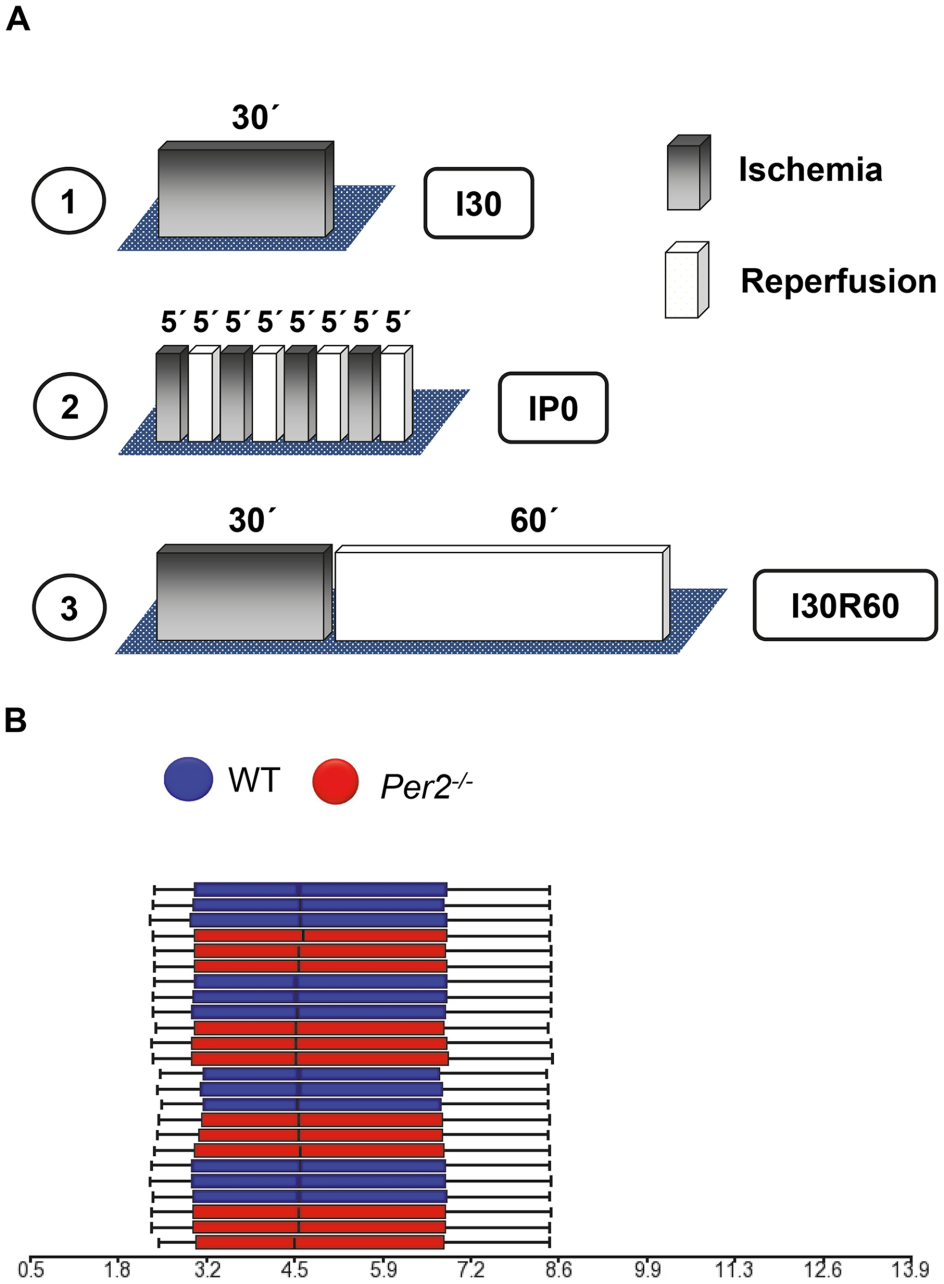
Microarray design comparing wildtype and *Per2* *^−^* ^*/−*^ mice. (**A**) Different ischemia and reperfusion protocols used on one 24 multi-plate array. 1.) 30 minutes of ischemia without reperfusion, I30. 2.) Ischemic preconditioning consisting of 4×5 minutes of ischemia followed by 5 minutes of reperfusion each, IP0. 3.) 30 minutes of ischemia and 60 minutes of reperfusion, I30R60. (**B**) Box plots for each of the samples with the intensity (arbitrary units) of the probes graphed on the X-axis to identify outliers in the data set.

**Figure 3:**
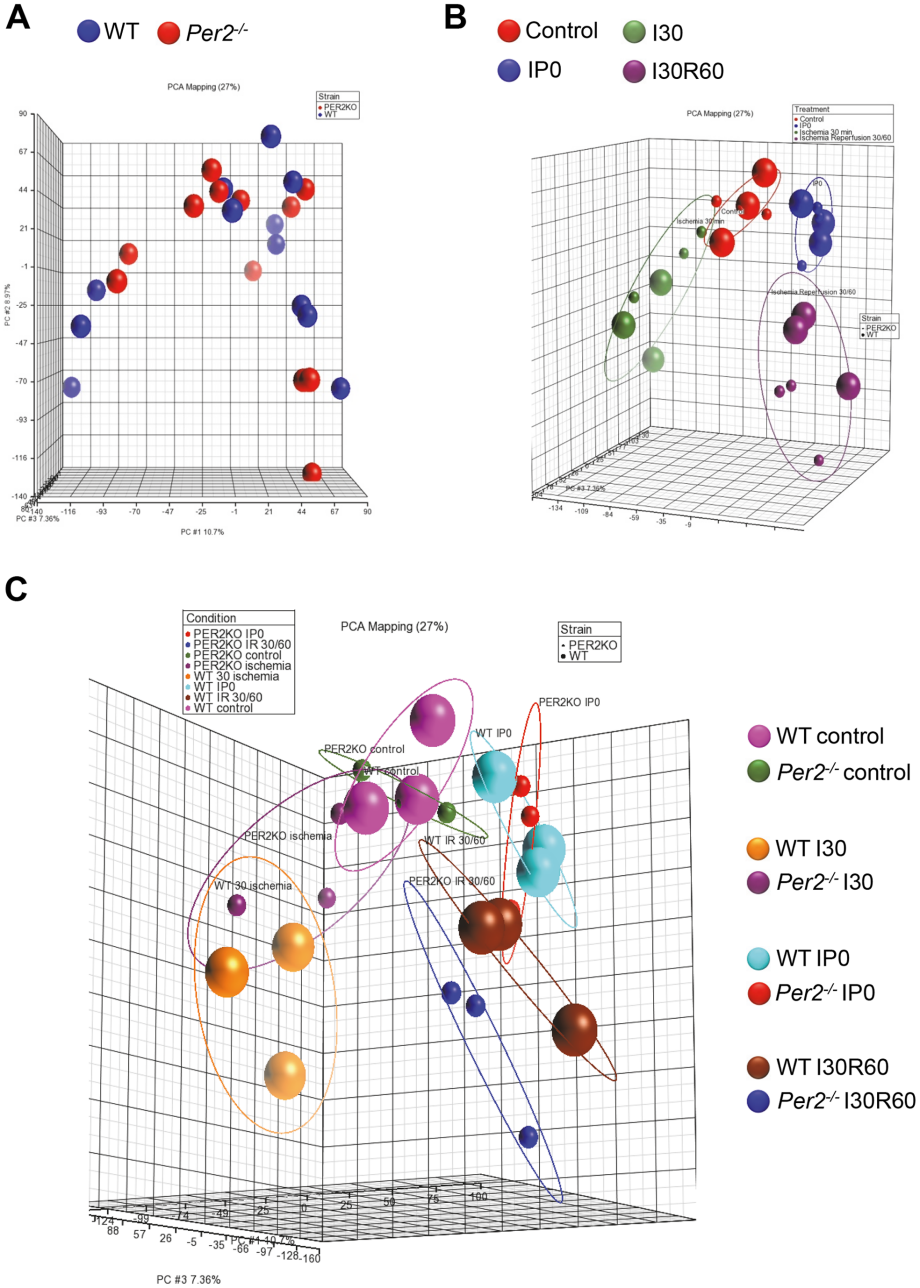
‘Principal Components Analysis’ (PCA) of a 24 multi-plate microarray. Each point represents a chip (sample) and corresponds to a row on the top-level spreadsheet. The color of the dot represents the type of the sample. Points that are close together within the plots have similar intensity values across the probe sets on the whole chip (genome), and points that are far apart within the plots are dissimilar. (**A**) PCA of the genetic background. (**B, C**) PCA of the different treatment conditions. WT =  wildtype, *Per2^−/−^*  =  Period 2 deficient mice, I30  = 30 minutes of ischemia without reperfusion, IP0  =  ischemic preconditioning (4×5 minutes of ischemia and reperfusion), I30R60  = 30 minutes of ischemia followed by 60 minutes of reperfusion. The units on the axes represent the different measurement points of all arrays where the percentage for one axis indicates how many of these measurement points are representable by this axis. *NOTE:* Due to the rotation of the 3-D graph using *Partek Genomics Suite 6.6* not all values are visible.

After confirming a dominant role for Per2 in regulating lactate metabolism during myocardial ischemia or reperfusion, we next pursued studies on Per2 dependent gene expression during myocardial ischemia or reperfusion to understand its impact on cardiac metabolism. We designed different ischemia and reperfusion protocols and performed high-throughput expression profiling of 24 samples at a time using an industry-standard whole mouse gene array (Affymetrix, Mouse Gene 1.1 ST 24-Array, **Figure** **2A**). To understand differential gene regulation during different conditions we performed 1) 30 minutes of ischemia without reperfusion (**Figure** **2A** **,** **1**), 2) ischemic preconditioning (IP, 4×5 minutes ischemia and reperfusion, **Figure** **2A, 2**), as a known cardioprotective mechanism, and 3) 30 minutes of ischemia followed by 60 minutes of reperfusion (**Figure** **2A** **,** **3**). As a control group sham operated hearts from wildtype or *Per2^−/−^* mice were used. Based on three arrays for each of these four conditions using two mouse strains, the total number of arrays was 24, which we analyzed at the same time on a multi plate array to avoid inter-array variations. Quality analysis using *Partek Genomics Suite 6.6* revealed high confidence in the quality of the microarray data and all samples met ‘Quality Assurance/Quality Control’ (QA/QC) criteria. As shown in **Figure** **2B**, box plots for each of the samples with the intensity of the probes graphed on the X-axis revealed the same distribution pattern indicating that there were no outliers in the data set. Next, we asked if similar samples resembled each other. Therefore, we performed a ‘Principal Components Analysis’ (PCA) as shown in **Figure** **3**. In the scatter plots, each point represents a chip (sample) and corresponds to a row on the top-level spreadsheet. The color of the dot represents the type of the sample. Points that are close together within the plots have similar intensity values across the probesets on the whole chip (genome), and points that are far apart within the plots are dissimilar. As seen in **Figure** **3A**, there was no clear separation between *Per2^−/−^* and wildtype samples just based on the genotype. However, if we clustered samples by different treatment conditions, data appeared clearly separated (**Figure** **3B and C**). As also seen in **Figure** **3B and 3C**, data became not only separated by treatment but also within treatment groups, revealing a separation of *Per2^−/−^* and wildtype samples (**Figure** **3C**). Being certain of the high quality of the array data, we next performed analysis of differentially regulated genes between wildtype and *Per2^−/−^*animals. As seen in **Table** **1** **,** ischemia without reperfusion (I30), ischemic preconditioning (IP0) or ischemia with reperfusion (I30R60), revealed 26, 31 or 52 differentially regulated genes in wildtype animals, respectively. Surprisingly, there were few genes regulated in *Per2^−/−^* mice when treated with ischemia or IP alone. In contrast, ischemia with reperfusion (I30R60) revealed 52 and 107 differentially regulated genes in wildtype and *Per2^−/−^* animals, respectively.

**Table 1:** Differentially regulated genes in wildtype compared to *Per2^−/−^* mice during myocardial ischemia and reperfusion.

*No of genes:*	*WT only*	*Common*	*Per2−/− only*
**Condition**			
I30	26	0	0
IP0	31	5	4
I30R60	52	113	107

Shown are the number of genes that were differentially regulated in wildtype or *Per2^−/−^* mice using different ischemia and reperfusion protocols: wildtype or *Per2^−/−^* mice were exposed to 1.) Ischemia of 30 minutes without reperfusion (I30), 2.) Ischemic preconditioning (consisting of 4 times 5 minutes of ischemia and 5 minutes of reperfusion, IP0), and 3.) 30 minutes of ischemia followed by 60 minutes of reperfusion (I30R60).

### Ischemia treatment reveals fatty acid metabolism as top Per2 dependent canonical pathway

**Figure 4:**
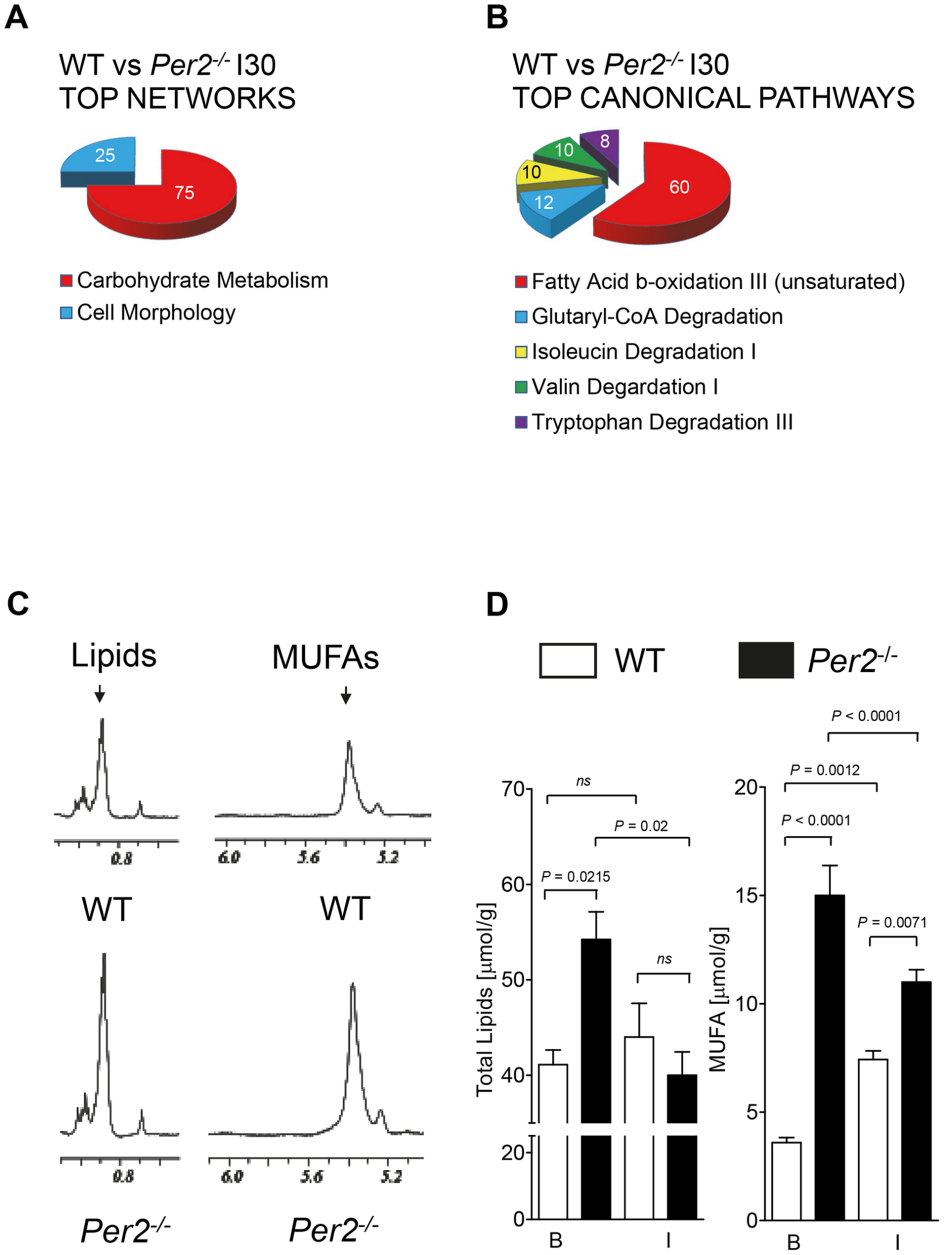
Disrupted fatty acid metabolism in *Per2* *^−^* ^*/−*^ mice during myocardial ischemia. (**A, B**) Pathway analysis comparing wildtype and *Per2^−/−^* mice after 30 minutes of ischemia without reperfusion. Differentially regulated genes and pathways were analyzed using Partek and Ingenuity software, respectively. (**C**) Fatty acid subpopulation analysis in wildtype and *Per2^−/−^* hearts at baseline using nuclear magnet resonance (NMR) technique. Representative NMR spectra for total lipids or monounsaturated fatty acids (MUFAs) at baseline are displayed. To calculate an absolute monounsaturated fatty acid (MUFA) concentration, the concentration of polyunsaturated fatty acids, triacylglycerides and glycerides are subtracted from the total for this peak. (**D**) WT or *Per2^−/−^* mice were exposed to 30 minutes of ischemia without reperfusion. Shock frozen hearts were analyzed for total lipid and MUFA content at baseline (B) and Ischemia (I) using NMR, n = 3 mice in all groups.

Next, differently regulated genes underwent pathway analysis using *Ingenuity Systems IPA* software. Analysis of genes regulated only in wildtype animals during ischemia revealed carbohydrate metabolism as top gene network (**Figure** **4A**). However, canonical pathway analysis showed a major role for Per2 in fatty acid beta-oxidation (**Figure** **4B**). Further analysis of top differentially regulated genes resulting in carbohydrate or fatty acid metabolism as the top network or top canonical pathway, uncovered a robust up regulation of protein phosphatase 1 (PP1) or down regulation of enoyl-CoA hydratase in wildtype animals with no regulation in *Per2^−/−^* mice (**Table** **2**). PP1 plays a crucial role in the regulation of blood-glucose levels and glycogen metabolism [38]. This is in line with earlier findings of severe depletion of and the inability to restore glycogen storages in *Per2^−/−^* mice during and after myocardial ischemia [25]. Enoyl-CoA hydratase is an enzyme that hydrates the double bond between the second and third carbons on acyl-CoA. This enzyme is essential to metabolizing fatty acids to produce both acetyl-CoA and energy. Inhibition of fatty acid beta-oxidation during ischemia is desirable as it helps the heart to be more oxygen efficient [39], [40]. Earlier studies on long chain fatty acids (LCFA) and carnitine palmitoyltransferase 1 (CPT1) in hearts from *Per2^−/−^* showed lower LCFA levels and increased CPT1 protein levels in *Per2^−/−^* mice [25], supporting the findings of the inability in *Per2^−/−^* to downregulate enoyl-CoA hydratase.

**Table 2:** Metabolism under the control of Per2.

Carbohydrate Metabolism ‘ WT genes’ I30	Fold change [Exp. Value]
surfactant protein C	6.63
***protein phosphatase 1, regulatory subunit 3C***	2.383
uncoupling protein 3	−3.14
**Lipid Metabolism ‘WT genes’ I30**	
surfactant protein C	6.63
activating transcription factor 3	3.489
***protein phosphatase 1, regulatory subunit 3C***	2.383
**enoyl-CoA, hydratase/3-hydroxyacyl CoA dehydrogenase**	−2.916
uncoupling protein 3	−3.14
**Carbohydrate Metabolism ‘ WT genes’ IP0**	
**nuclear receptor subfamily 4, group A, member 1**	3.017
**nuclear receptor subfamily 4, group A, member 2**	2.727
***protein phosphatase 1, regulatory subunit 3C***	2.514
**protein phosphatase 1, regulatory subunit 15A**	2.303
**Lipid Metabolism ‘WT genes’ IP0**	
FBJ murine osteosarcoma viral oncogene homolog	6.122
**nuclear receptor subfamily 4, group A, member 1**	3.017
**nuclear receptor subfamily 4, group A, member 2**	2.727
heat shock 70kDa protein 8	2.552
natriuretic peptide B	2.348
***protein phosphatase 1, regulatory subunit 3C***	2.514
**enoyl-CoA, hydratase/3-hydroxyacyl CoA dehydrogenase**	−2.072
**Carbohydrate Metabolism ‘ WT genes’ I30R60**	
interleukin 1, beta	3.556
**nuclear receptor subfamily 4, group A, member 3**	3.354
neurotensin	3.128
**nuclear receptor subfamily 4, group A, member 2**	2.742
**nuclear receptor subfamily 4, group A, member 1**	2.576
solute carrier family 5 (sodium/myo-inositol cotransporter), member 3	2.492
connective tissue growth factor	2.112
**Lipid Metabolism ‘WT genes’ I30R60**	
prostaglandin-endoperoxide synthase 2	4.15
**nuclear receptor subfamily 4, group A, member 3**	3.354
**nuclear receptor subfamily 4, group A, member 2**	2.742
**enoyl-CoA, hydratase/3-hydroxyacyl CoA dehydrogenase**	−2.012

Shown are the top metabolic genes accounting for the identification of carbohydrate or fatty acid metabolism as top networks or canonical pathways when analyzing genes that are only regulated in wildtype but not in *Per2^−/−^* mice using different ischemia and reperfusion protocols. WT  =  wildtype, I30  = 30 minutes of ischemia, IP0  =  ischemic preconditioning (4 times 5 minutes of ischemia and reperfusion), I30R60  = 30 minutes of ischemia and 60 minutes of reperfusion. Given are the expression values (fold change) obtained by Ingenuity pathway analysis. **Bold genes** appear in more than one treatment group, indicating a robust differentially regulated gene.

**Figure 5:**
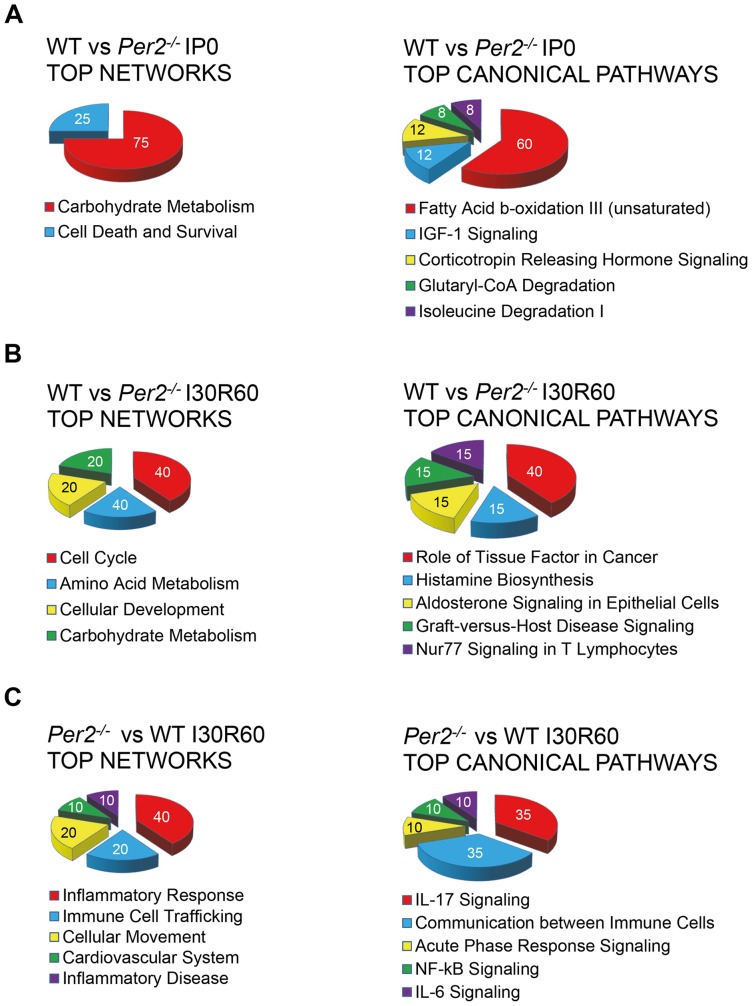
Ingenuity pathway analysis in wildtype and *Per2* *^−^* ^*/−*^ after IP or IR treatment. (**A**) Top networks or canonical pathways from differentially regulated genes after ischemic preconditioning (4×5 minutes of ischemia and reperfusion, IP0) treatment. Analysis is based on genes regulated in wildtype mice only. (**B**) Top networks or canonical pathways from differentially regulated genes after 30 minutes of ischemia and 60 minutes of reperfusion (I30R60) treatment, comparing wildtype and *Per2^−/−^* mice. Analysis is based on genes regulated in wildtype mice only. (**C**) Top networks or canonical pathways from differentially regulated genes after ischemia and reperfusion (I30R60) treatment comparing *Per2^−/−^* and wildtype mice. Analysis is based on genes regulated in *Per2^−/−^* mice only.

Metabolism of saturated and unsaturated fatty acids converges on the level of trans-2-enoyl-CoA, which is metabolized by enoyl-CoA hydratase. The inability of *Per2^−/−^* mice to down regulate enoyl-CoA hydratase during ischemia therefore implies changes in cardiac fatty acid levels. To gain insight into fatty acid subpopulations in *Per2^−/−^* hearts, we next performed metabolic analysis of heart tissue exposed to 30 minutes of ischemia using nuclear magnet resonance technique (NMR). In contrast to earlier findings on LCFA in hearts from *Per2^−/−^* mice [25], these studies revealed a significantly higher total lipid content in hearts from *Per2^−/−^* at baseline. Representative NMR spectra at baseline are displayed in **Figure** **4C**. As shown in **Figure** **4D** total cardiac lipid concentrations at baseline were in wildtype 41.4 µmol/g (SD2.6) and in *Per2^−/−^* 54.2 µmol/g (SD5.0) [*P* = 0.0215, n = 3]. Subsequent analysis of subpopulations revealed that *Per2^−/−^* had significantly higher monounsaturated fatty acid (MUFA) levels (**Figure** **4C, D**) accounting for this finding (Baseline (B): wildtype: 3.6 µmol/g (SD0.4) vs. *Per2^−/−^*:15.0 µmol/g (SD2.4), *P*<0.0001, n = 3). In line with studies on a Per2 dependent inhibition of fatty acid beta-oxidation [24], total lipid and MUFA levels significantly decreased during ischemia in *Per2^−/−^* (Total lipids/MUFA Baseline (B) *Per2^−/−^*: 54.2 µmol/g (SD5.0) /15.0 µmol/g (SD2.4) vs. Ischemia (I) *Per2^−/−^*: 40.0 µmol/g (SD4.2)/ 11.0 µmol/g (SD1.0), *P* = 0.02/*P*<0.0001, n = 3). In contrast, total lipids and MUFA levels increased in wildtype animals during ischemia, supporting the idea of inhibited fatty acid beta-oxidation as a protective mechanism (MUFA Baseline (B) wildtype :3.6 µmol/g (SD0.4) vs. Ischemia (I) wildtype: 7.4 µmol/g (SD0.6), *P* = 0.0012, n = 3). Indeed, analysis of genes regulated in wildtype animals only after IP treatment, a powerful cardioprotective mechanism [41], revealed identical networks, pathways and top genes as seen with 30 minutes of ischemia alone (**Figure** **5A**). Taken together, studies comparing ischemic hearts from *Per2^−/−^* and wildtype mice reveal a dominant role for cardiac Per2 as regulator of lipid metabolism and uncover enoyl-CoA hydratase as putative Per2 target gene in the heart.

### Reperfusion regulated genes in *Per2^−/−^* mice resemble a strong pro-inflammatory phenotype

After identification of Per2 as an important regulator of lipid metabolism during ischemia, we next analyzed differentially regulated genes during reperfusion. While genes regulated only in wildtype animals mainly consisted of cell cycle or metabolic genes (**Figure** **5B**), genes only regulated in *Per2^−/−^* mice revealed the activation of a robust pro-inflammatory program. As seen in **Figure** **5C**, top networks or top canonical pathways were dominated by immune cell trafficking, IL-17/IL-6, or NF-κβ signaling. Details on top genes up regulated in *Per2^−/−^* mice during reperfusion are given in **Table** **3**. Following review of current literature on these genes confirmed severe pro inflammatory action and in part detrimental roles in cardiovascular disease [42]–[45]. Taken together, analysis of differentially regulated genes during reperfusion reveal a severe pro-inflammatory phenotype in *Per2^−/−^*.

**Table 3:** Inflammation under the control of Per2 during myocardial ischemia and reperfusion.

Inflammatory ‘Per2 genes’ I30R60	Fold change [Exp. Value]
Resistin-like molecule-beta	8.02
CCL3L1/CCL3L3	7.855
Metalloproteinase-8	5.05
Tumor Necrosis Factor	4.286
Immunoresponsive gene 1	4
Gadd45beta	3.9
S100A8/A9	3.919
IL1A interleukin 1, alpha	2.991
Interleukin 36, gamma	2.718
Triggering receptor expressed on myeloid cells 1	2.386
Toll-like receptor 2	2.212
Interleukin 17 receptor A	2.2
IL1R1 interleukin 1 receptor, type I	2.112

Shown are the top genes accounting for the identification of a dominant pro inflammatory program when analyzing genes that are only regulated in *Per2^−/−^* mice using 30 minutes of ischemia and 60 minutes of reperfusion. Given are the expression values (fold change) obtained by Ingenuity pathway analysis. I30R60  = 30 minutes of ischemia and 60 minutes of reperfusion.

### Reperfusion injury in *Per2^−/−^* is associated with increased inflammatory cytokines

**Figure 6:**
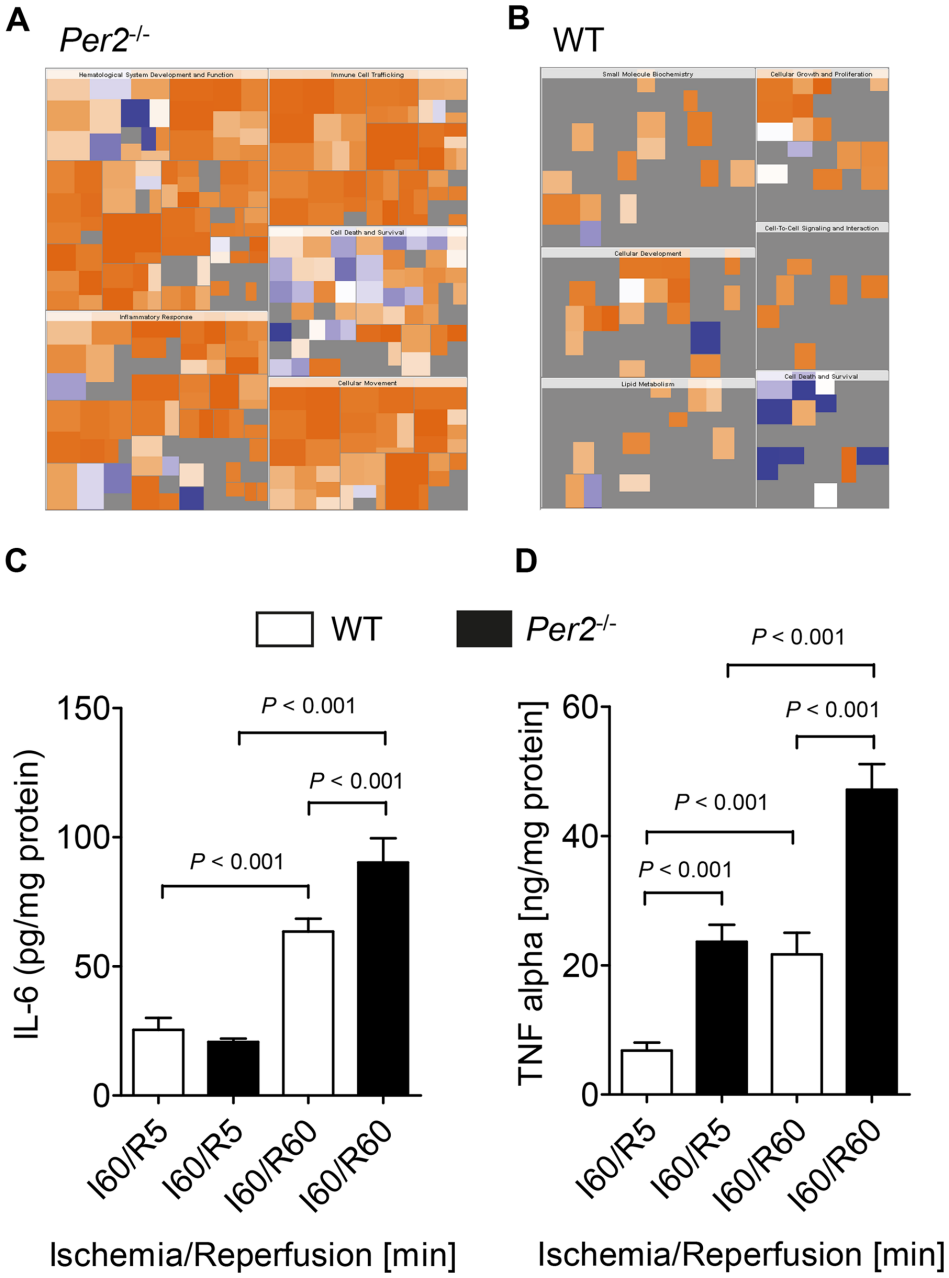
Initiation of a pro- inflammatory program in *Per2* *^−^* ^*/−*^ mice during ischemia and reperfusion. (**A,B**) Pattern recognition analysis (heat map of biological functions) from genes only regulated in *Per2^−/−^* (**A**) or WT (**B**) mice after 30 minutes of ischemia and 60 minutes of reperfusion. (**C, D**) Wildtype or *Per2^−/−^* mice were exposed to 60 minutes of ischemia and 5 (I60/R5) or 60 (I60/R60) minutes of reperfusion. The area at risk was excised and analyzed for IL-6 (**C**) or TNF-α (**D**) cardiac tissue concentration; n = 3 mice in all groups.

After uncovering an unexpected role of Per2 in controlling inflammation during myocardial ischemia and reperfusion, we next performed a pattern recognition analysis (heat map of biological functions) of differentially regulated genes in *Per2^−/−^* and wildtype mice. As seen in **Figure** **6A**, heat map analysis revealed a very strong focus on pro-inflammatory pathways, indicated by the deep orange color. The intensity of the orange color indicates “activated” or “increased function or disease”. In contrast, the blue color reflects “inhibited” or “decreased function or disease.” These pro-inflammatory pathways in *Per2^−/−^*, as seen in **Figure** **6A**, consisted of biological functions such as ‘hematologic system development’, ‘immune cell trafficking’, ‘inflammatory response’, ‘cellular movement’ and ‘cell death and survival’. In contrast, heat map analysis from wildtype mice (**Figure** **6B** **)** revealed ‘small molecule biochemistry’, ‘cellular growth and proliferation’, ‘cellular development’, ‘lipid metabolism’, ‘cell to cell signaling and interaction’ and ‘cell death and survival’ as major biological functions. However, while the heat map in *Per2^−/−^* mice indicated a very strong activation of the inflammatory response (dark orange), we did not find a similar ‘hot spot’ in any of the biological functions associated with the wildtype mice (**Figure** **6B** **)**. Next, to confirm the data obtained from the array we studied cardiac cytokine levels during reperfusion after 60 minutes of ischemia in *Per2^−/−^* and wildtype mice. As seen in **Figure** **6C and D**, IL-6 and TNF-α levels increased during 60 minutes of reperfusion in wildtype and *Per2^−/−^* animals. While IL-6 was only significantly higher in *Per2^−/−^* mice at 60 minutes of reperfusion (63.5 pg/mg (SD8.4) vs. 90.24 pg/mg (SD16.17) in wildtype or *Per2^−/−^*, respectively, *P*<0.001, n = 3), TNF-α levels were significantly higher in *Per2^−/−^* at 5 and 60 minutes of reperfusion (wildtype I60/R5: 6.8 ng/mg (SD2.1) vs. *Per2^−/−^*: I60/R5, 23.6 ng/mg (SD4.5), *P*<0.001, wildtype I60/R60: 21.7 ng/mg (SD5.8) vs. *Per2^−/−^*: 47.1 ng/mg (SD6.8), *P*>0.001, n = 3). Taken together, these data suggest a strong anti-inflammatory role of Per2 during myocardial ischemia and reperfusion.

## Discussion

The question addressed in this study was to understand the contribution of Per2 to cardiac metabolism during myocardial ischemia and reperfusion. The main finding of our study is that Per2 activation during ischemia regulates fatty acid beta-oxidation during ischemia and inflammation during reperfusion.

Studies on cardiac lactate production during myocardial ischemia confirm a non-redundant role of Per1 and Per2 for cardiac metabolism, where lactate production is linked to Per2 but not Per1. Following detailed microarray analysis, we identified protein phosphatase 1 (PP1) as putative Per2 target gene, which is in line with earlier findings on a role of Per2 for regulating cardiac glycogen levels. In contrast, top canonical pathway analysis showed Per2 as important control point in fatty acid beta-oxidation and suggests enoyl-CoA hydratase as an extremely robust Per2 target gene in the heart. Nuclear magnetic resonance studies further confirmed a regulatory role for Per2 in cardiac fatty acid metabolism during myocardial ischemia. Finally, studies during reperfusion after 60 minutes of myocardial ischemia found a strong, so far unknown, anti-inflammatory role for cardiac Per2.

Fifteen-20 seconds after the occlusion of coronary vessels, anaerobic glycolysis supervenes as the only significant source of new high-energy phosphate. This is sufficient to meet at least the most basic energy demand of cardiomyocytes, however within 60 to 90 minutes of ischemia the affected area of the heart develops contracture-rigor [46]. If anaerobic glycolysis is inhibited, in less than five minutes, the reserve supplies of energy phosphates are depleted totally and the heart undergoes contracture-rigor [46]. These ‘simple’ experiments support our recent findings where *Per2^−/−^* are incapable of lactate production during myocardial ischemia and therefore have larger infarct sizes. In addition, findings on Per2 as an exclusive regulator of anaerobic glycolysis or lactate production when compared to Per1, indicate an important role in controlling cardiac metabolism under these pathologic conditions.

In contrast, it is well known that anaerobic glycolysis or lactate production is under the control of hypoxia inducible factor 1 (Hif1α[47]. Indeed, recent studies in *Per2^−/−^* mice found not only a lack of lactate production during ischemia, but also a deficiency in Hif1α dependent regulation of glycolytic enzymes. Moreover, co-immunoprecipitation studies confirmed a co-localization of both Per2 and Hif1α in the nucleus during myocardial ischemia [25]. Although Per2 cannot bind to DNA itself, it has been shown that Per2 is able to act as co- regulator of transcription [48]. The findings from our microarray analysis in the current study support this concept: microarray analysis from ischemic hearts without reperfusion revealed almost no regulation of transcripts in *Per2^−/−^*. This suggests that Per2 is particularly important in controlling transcription under pathologic conditions such as myocardial ischemia.

Glucose and glycogen metabolism are interlinked. Under conditions of total global ischemia, glycogen is the only substrate for glycolytic flux. As such, glycogen regulation is a critical mechanism for the heart during myocardial ischemia and reperfusion. *Per2^−/−^* mice show a severe depletion of glycogen content during ischemia and are not able to recover glycogen storages during reperfusion [25]. The findings on Per2 dependent regulation of PP1, an important regulator of glycogen metabolism, support these findings. Earlier studies have elegantly pointed out that activation of glycogen synthesis is associated with an increase in PP1 activity [49]. Interestingly another study on hypoxic preconditioning of isolated hearts, as model for cardioprotection, found PP1 as a mediator of a PKC-independent protection on ischemic-reperfused cardiomyocytes [50]. This is in line with our findings on ischemic preconditioning (IP) of in situ perfused hearts that show the up regulation of PP1 in wildtype mice only. In fact, earlier studies demonstrated that *Per2^−/−^* are not protected by IP [25].

The circadian control of cardiac lipid metabolism has been pointed out by elegant studies from a group led by Young et al. For example, heart specific Clock mutant mice directly regulate myocardial triglyceride metabolism [22]. Moreover, gene expression studies suggested that the cardiomyocyte circadian clock influences myocardial contractile function, metabolism, and gene expression [51]. A recent study, using mice with an adipocyte-specific deletion of Bmal1, a gene encoding a core molecular clock component, found obesity and reduced numbers of polyunsaturated fatty acids in adipocyte triglycerides [23]. Another study using Per2-deficient mice, found altered lipid metabolism with drastic reduction of total triacylglycerol and non-esterified fatty acids. In contrast, we found recently an important role of Per2 for carbohydrate metabolism during myocardial ischemia [25]. However, as glycolysis and lipid metabolism are interlinked and peripheral ‘clocks’ seem to be dominant regulators of lipid metabolism in general, it is compelling that Per2 might play a similar role in the heart. This concept is strongly supported by our microarray analysis looking at differentially regulated genes between wildtype and *Per2^−/−^*. Here we found lipid metabolism as the top canonical pathway during ischemia or IP without reperfusion. Moreover, in all conditions analyzed (24 arrays), enoyl-CoA hydratase was only regulated in wildtype animals. This robust phenotype stresses the importance of circadian rhythm proteins in regulating fatty acids in general and uncovers a novel role for Per2 in cardiac fatty acid beta-oxidation. Whether Clock and Per2 have similar roles, are interacting or have different functions will require future studies.

Recent findings strongly suggest an important role of the circadian clock in innate and adaptive immunity [52]–[54]. For example, one study found a role for Clock in controlling Toll-like receptor-9 mediated inflammation (49). Interestingly, several studies have already shown the importance of toll like receptors and innate immunity for myocardial ischemia and reperfusion [55]–[57]. Other studies have reported a link between the circadian clock and TNF-α [58]. However, to our knowledge, a circadian control of inflammation in the heart during myocardial ischemia and reperfusion has not been described. In the current study, our microarray screen found the activation of a very strong pro-inflammatory program during myocardial ischemia and reperfusion in *Per2^−/−^* mice. Studies on TNF-α and IL-6 during reperfusion confirmed these findings. As *Per2^−/−^* have bigger infarct sizes than wildtype mice [25], more invading inflammatory cells into a more severely damaged myocardium could possibly explain higher cytokine levels in *Per2^−/−^* mice. However, recent published studies on myocardial ischemia in wildype and *Per2^−/−^* mice showed significant infarct sizes in both mice [25]. Therefore, the finding of a very distinct pro-inflammatory gene pattern, upregulated in *Per2^−/−^* mice only, cannot fully be explained by larger infarct sizes in *Per2^−/−^*. In fact, complete lack of these transcripts in wildtype mice indicate that Per2 is a necessary suppressor. However, if the anti-inflammatory function of Per2 is mediated by its function in the vasculature [59], myocytes or the invading inflammatory cells [60] will require future studies using conditional knockout mouse models.

In general, these data in conjunction with findings on metabolism are in favor of the idea that metabolism and inflammation are connected and that inflammation can be a consequence of pathologic metabolism [61]. In fact, disruption of circadian protein pathways has been shown to lead to a phenotype in mice that resembles the metabolic syndrome found in human subjects [12], [15], [16], [62]. Interestingly, patients who have a metabolic syndrome and have higher inflammatory markers are at greater risk to develop cardiovascular disease [63]. Therefore, understanding the systems linking circadian rhythmicity to cardiac cell-metabolism and cardiac cell-inflammation could prove useful insights into ischemic heart disease.

In summary, performing a high throughput gene array screen in hearts from *Per2^−/−^* in conjunction with analysis of metabolism and inflammation unveil a novel role for Per2 in fatty acid beta-oxidation and inflammation during myocardial ischemia and reperfusion, respectively. If confirmed by future studies in animals or human subjects, this could lead to the discovery of new therapeutic concepts in myocardial ischemia.
